# Lingual hamartoma‐like lipoblastoma: the diagnostic value of routine whole‐genome sequencing

**DOI:** 10.1111/his.15457

**Published:** 2025-04-13

**Authors:** Sheng‐Yuan Kan, Ashley Ferro, James A Watkins, Sam Behjati, Matthew J Murray, Nicholas Coleman, Thomas Roberts, Justin Cross, Jamie Trotman, Patrick Tarpey, C Elizabeth Hook, Malcolm Cameron, John A. Tadross

**Affiliations:** ^1^ Department of Histopathology Cambridge University Hospitals NHS Foundation Trust Cambridge UK; ^2^ Department of Oral and Maxillo‐Facial Surgery Cambridge University Hospitals Cambridge UK; ^3^ East Genomic Laboratory Hub (GLH) Genetics Laboratory Cambridge University Hospitals NHS Foundation Trust Cambridge UK; ^4^ Department of Paediatric Haematology and Oncology Cambridge University Hospitals NHS Foundation Trust Cambridge UK; ^5^ Wellcome Sanger Institute Cambridge UK; ^6^ Department of Pathology University of Cambridge Cambridge UK; ^7^ Department of Radiology Cambridge University Hospitals NHS Foundation Trust Cambridge UK; ^8^ Medical Research Council Metabolic Diseases Unit Institute of Metabolic Science Metabolic Research Laboratories, University of Cambridge Cambridge UK

**Abbreviations**
FISH: Fluorescent *in situ* hybridisation
NHS: National health service
WGS: whole genome sequencing

Paediatric tongue lesions are rare, and diagnostically challenging as a result.^1^ Histopathology often suffices for diagnosis, but some lesions require molecular assessment for precise characterization. All patients under 25 years of age with a differential that includes neoplasia are eligible for whole‐genome sequencing (WGS) on the NHS, and widespread adoption has the potential to disrupt routine pathology practice by providing comprehensive genomic characterization within days.^2^, ^3^, ^4^ Here we describe a 2‐year‐old with a tongue lesion that highlights the diagnostic value of routine WGS.

## Case report

**Figure 1:**
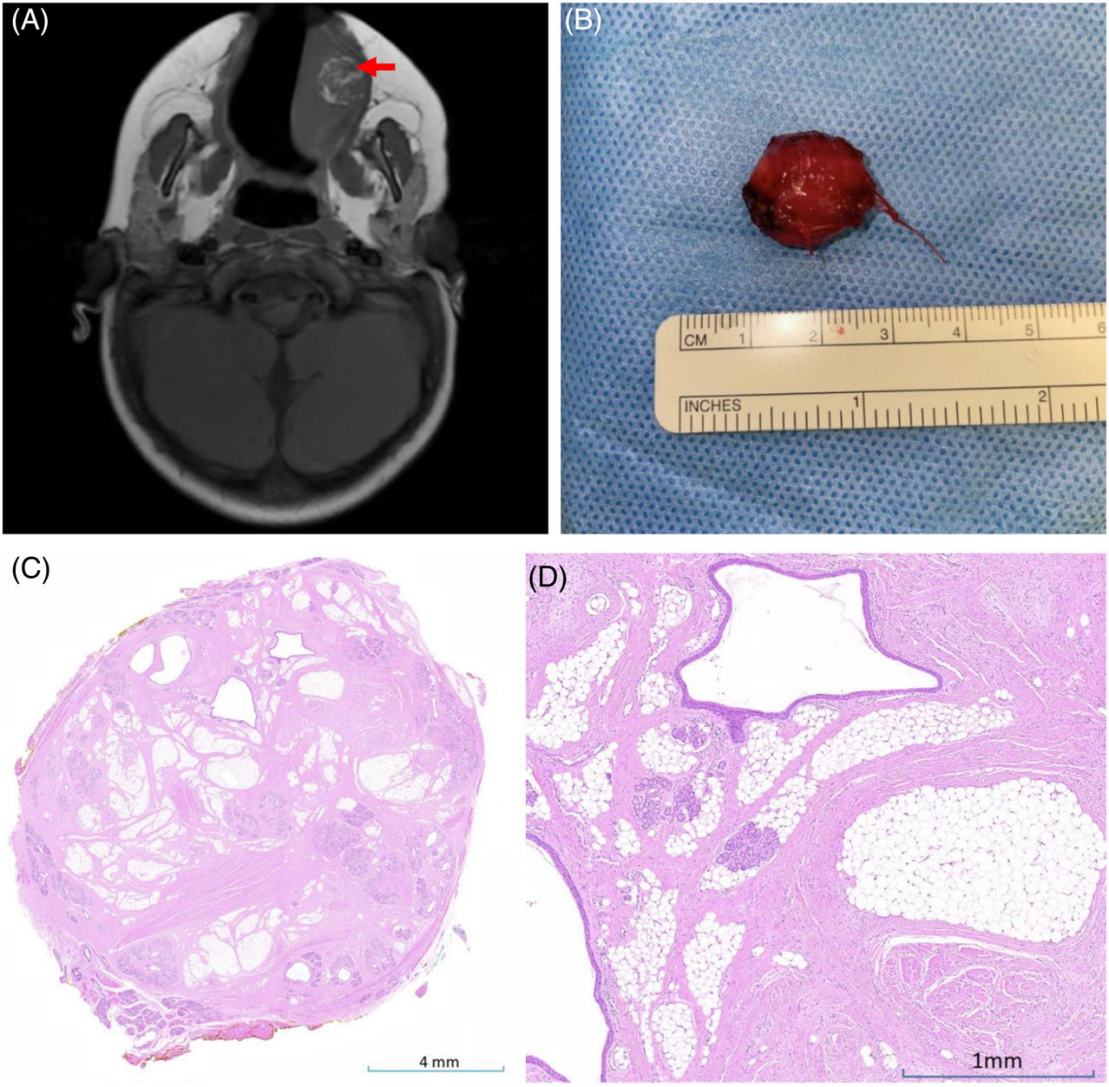
**(A)** Baseline imaging with T1‐weighted axial MRI precontrast showing the lesion (red arrow). **(B)** Macroscopic image of the resected tumour. **(C,D)** Haematoxylin and eosin (H&E)‐stained sections demonstrating fibroconnective tissue and adipose tissue centrally, admixed distorted skeletal muscle, lobules of salivary glands, and ectatic ducts. [Color figure can be viewed at wileyonlinelibrary.com]

A 2‐year‐old girl presented with a 2‐week history of left tongue swelling, irritability, and drooling. She was otherwise healthy, with no feeding or respiratory issues. Examination revealed a 2‐cm diameter submucosal swelling. Magnetic resonance imaging (MRI) confirmed a 1.5‐cm diameter well‐defined lesion with nodular high‐intensity areas on T1 and moderate gadolinium enhancement, suggestive of a lipoma or hamartoma, with liposarcoma as a differential (Figure 1A). The lesion was surgically excised, appearing as an encapsulated nodule (Figure 1B). Histological examination revealed a mixture of fibrous and adipose tissues, atrophic skeletal muscle, and disorganized salivary glandular elements (Figure 1C,D). The initial diagnosis, based on the consensus of one paediatric and two head and neck pathologists, was of a benign lingual hamartoma.

**Figure 2:**
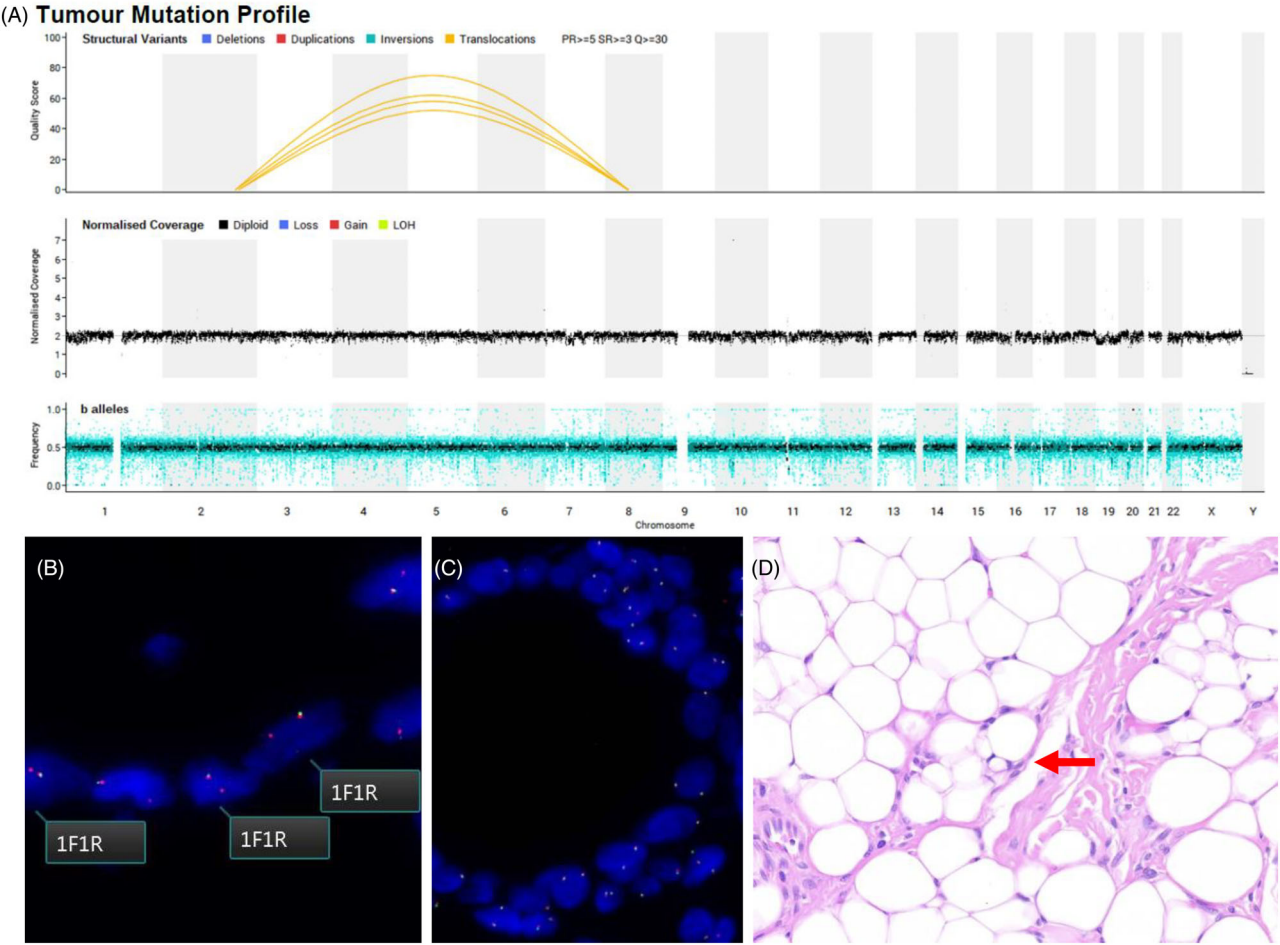
**(A)** Whole‐genome sequencing showing a quiescent genome with a single t(2;8) *COL3A1::PLAG1* driver event. **(B)** FISH showing adipocytes with predominantly one fused (F) and one red probe (R) signal, indicating monoallelic loss of the 5′ region of the hybridized PLAG1 locus. **(C)** The epithelial component shows two fused signals with no evidence of PLAG1 break apart. **(D)** Sparse lipoblasts are seen on review. [Color figure can be viewed at wileyonlinelibrary.com]

Fresh tissue from the lesion was submitted for WGS, which is routine practice in our hospital for all potential neoplasia in consenting patients under 25 years of age. Unexpectedly, WGS identified an in‐frame *COL3A1::PLAG1* fusion (Figure 2A), which was subsequently confirmed by RNA fusion testing. This fusion is recurrent in lipoblastoma,^5^ and *PLAG1* is also the most frequent fusion partner in pleomorphic adenoma (PA), a common salivary gland neoplasm that can exhibit both epithelial and mesenchymal differentiation. It is conceivable that a PA could present with lingual hamartoma‐like features, and data on the molecular landscape of lingual hamartomas is limited. We reasoned that were this an unusual PA rather than lipoblastoma, the epithelial and mesenchymal components would harbour the *PLAG1* translocation. Fluorescence *in situ* hybridisation (FISH) analysis demonstrated that the rearrangement was restricted to adipocytes (Figure 2B,C), and the epithelial cells were nonneoplastic. Upon reexamination of the histology, subtle lipoblasts were noted interspersed among predominantly mature adipocytes (Figure 2D).

The patient recovered well postsurgery and, given the diagnosis, remains under close surveillance due to the risk of recurrence.^3^

## Discussion

Paediatric oral lesions often present nonspecific clinical and imaging features, complicating the differential diagnosis.^1^ In this case, the absence of immature lipoblasts and myxoid areas initially led to the exclusion of a lipoblastoma diagnosis.^6^ WGS, however, detected a pathognomonic *COL3A1::PLAG1* fusion, underscoring its diagnostic utility in ambiguous cases. Lipoblastomas, unlike hamartomas, carry a risk of local recurrence and therefore necessitate closer postoperative surveillance.^7^

Routine WGS, as integrated into clinical practice by NHS England through initiatives such as the 100,000 Genomes Project, offers a powerful tool for comprehensive genomic assessment, substantially enhancing diagnostic accuracy, and informing treatment strategies. The rapid turnaround of WGS data will make it contemporaneously available with histological findings, allowing for seamless integration into diagnostic reports.^1^, ^2^ This has profound implications for histopathology, providing orthogonal diagnostic feedback, highlighting discrepancies (as in this case), and ultimately leading to better patient outcomes.

## Author contributions

S.‐Y.K., A.F., and J.A.T. wrote the article and prepared the figures. J.A.W., S.B., M.J.M., T.R., P.T., J.T., C.E.H., and J.A.T., interpreted the whole‐genome sequencing data. All authors contributed to the conceptualisation, authorship, and review of the article.

## Funding information

S.B. acknowledges funding from the Wellcome Trust (institutional grant; personal fellowships; grant number 108413/A/15/D and 223135/Z/21/Z). J.A.T. is supported by the MRC Metabolic Diseases Unit (MC_UU_00014/1).

## Conflict of interest

The authors have nothing to declare.

## Data Availability

As per Genomics England whole‐genome sequencing consent, we are unable to share raw sequencing data for the patient described. Nevertheless, researchers may apply to join the Genomics England Research Network to access anonymised sequencing data and a summary of findings submitted by the Genomic Laboratory Hub.
